# A Case of Myoma of the Cervix Uteri

**Published:** 1885-11-20

**Authors:** Virgil O. Hardon

**Affiliations:** Atlanta, Ga.


### Southern Medical Record.

EDITORS:

THOMAS S. POWELL, M.D. R. C. WORD, M.D.

*R. C. WORD, M.D., Managing Editor.*

☛ All Communications and Letters on Business connected with the RECORD must be addressed to the Managing Editor.

VOL. XV. ATLANTA, GA., NOVEMBER 20, 1885. NO. 11.

### ORIGINAL AND SELECTED ARTICLES.

### A CASE OF MYOMA OF THE CERVIX UTERI.

BY VIRGIL O. HARDON, M. D., ATLANTA, GA.

N. W——, white ; aged twenty-five ; married ; first menstruated at seventeen years of age, and became regular after two months. The flow was profuse and painless, lasting five days. She was married at eighteen, and bore one child thirteen months after marriage. Her menses returned in five months, and no symptoms of uterine trouble followed. Three years later she miscarried at four months. Since that time she has been very irregular in her menstruation, suffering at different times from menorrhagia, metrorrhagia and amenorrhœa. Since her miscarriage she has had constant pain in her back, hips, and lower part of the abdomen. The urine has been passed frequently and with pain. A profuse leucorrhœa has also existed.

Patient is pallid and anæmic ; bowels regular, appetite poor. She was first seen by me in July, at which time she had not menstruated for two months. Vaginal examination showed the uterus to be sharply anteflexed and enlarged. The angle of anteflexion was situated about at the internal os. There was a slight laceration on the right side of the cervix. In the posterior lip, about a half an inch from the os and just below the cervico-vaginal junction, was a fistula large enough to admit the little finger, extending through the lip from the cervical canal to the vagina. The edges ef this fistula were composed of hard cicatricial tissue. At this fistula, and partially projecting through it, a tumor presented itself, composed of a dense fibrous structure, and attached to the inner surface of the anterior cervical wall. This tumor extended upward, completely obstructing the internal os so that a probe could not be passed by it. A sound inserted at the external os emerged through the fistula. There was some tenderness in the roof of the pelvis surrounding the womb.

The patient was placed upon tonics, and ordered to take as much fresh air, exercise and sunlight as possible. She was also ordered to use hot vaginal injections twice a day, and Churchill's tincture of iodine was applied to the whole vaginal surface twice a week. The menses occurred in August and September, with normal amount and duration, but accompanied by severe pain. The tenderness in the roof of the pelvis had entirely disappeared, and the patient was much improved in her general condition.

On Sunday, October 4, the patient was etherized and the posterior lip of the cervix was divided up to the fistula. The tumor, which grew by a broad base from the anterior cervical wall, was removed by curved scissors. It extended upward into the internal os, but did not invade the body of the womb, as was determined by exploration with the finger. The growth was of the size of a walnut, and extended deep into the normal tissues of the cervix. There was no definite line of demarcation between the growth and the normal tissues. The tumor was dense, and was cut with difficulty, creaking under the scissors. It was not deemed advisable to reunite the posterior lip by sutures as the loss of tissue by reason of the fistula would have produced a stenosis of the cervical canal at that point after its edges were brought together.

The operation was performed with strict antiseptic precautions, the instruments, sponges, and other appliances, as well as the hands of the operator and assistants, being thoroughly carbolized. After the operation, the vagina was syringed out with a 2-per cent. carbolic solution, twice a day.

Patient made a good recovery, and menstruated two weeks after the operation without pain. The anteflected uterus was restored to its proper position by a Simpson's sound, and retained there by the use of the vaginal tampon of cotton. The leucorrhœa has ceased, and there has been no return of the pain and tenderness in the pelvis. She has improved very much in general condition since the operation.

A portion of the tissue removed was submitted to Dr. Henry Wile for microscopic examination, and was found to be composed of unstriped muscular fibres. As a result of his report, a diagnosis was made of myoma of the cervix uteri. The condition is described by Klebs as "partial hyperplasia of the uterine parenchyma," of which he says: "Microscopic investigations show that the chief mass of the tumor consists of smooth muscular fibres, which considerably exceed in size those of the unimpregnated uterus. The muscular fibres are arranged in bundles, and the latter unite variously at acute angles to form larger groups, which enclose a wide capillary blood vessel. Between the muscular bundles, as well as between these and the connective tissue sheaths of the vessels, may be observed everywhere, by careful treatment, narrow slit-like gaps, which here and there contain white blood corpuscles. A cavernous structure thus originates, which is not found in the normal uterine tissue. Differences in the structure of these tumors are occasioned by the preponderating development of one of their histological constituents. A preponderating development of the muscular tissue, which would stamp the tumors as pure myomata, is rare; in general, the formation of the muscular substance runs parallel with the vascular development, and the richer nutritive supply thereby originating; yet tumors also occur which, from the outset, consist almost entirely of smooth muscular fibres, possess the grayish-red, dimly transparent appearance of normal uterine muscular tissue, and are evidently contractile.*"

